# The burden of substance use and (mental) distress among asylum seekers: a cross sectional study

**DOI:** 10.3389/fpsyt.2023.1258140

**Published:** 2023-12-20

**Authors:** Maximilian Solfrank, Christoph Nikendei, Catharina Zehetmair, Hans-Christoph Friederich, Ede Nagy

**Affiliations:** Department of General Internal Medicine and Psychosomatics, University Hospital Heidelberg, Heidelberg, Germany

**Keywords:** asylum seekers, refugee, substance use disorders, mental health, risk and protective factors

## Abstract

**Background:**

Asylum seekers are a particularly vulnerable population due to a wide range of external stressors. Traumatic events and difficult social/economic prospects can lead to an elevated susceptibility for substance use disorders. The aim of the present study was to determine whether asylum seekers suffering from mental or physical distress present higher levels of substance use disorder (SUD) in a state reception center in Germany and whether there are identifiable risk or protective factors.

**Methods:**

We performed a hierarchical logistic regression on data of *N* = 238 people who had applied for asylum in Germany to analyze the SUD variance explanation by (1) sociodemographic, (2) flight-specific, and (3) psychometric (ERQ, SOC-9 L, SCL-K9) variables. On level (4), we included the location of data collection (walk-in clinic or accommodation,) as an indicator of individual’s need for a psychologist’s or General practitioner’s help in order to assess for the participant’s (mental) distress.

**Results:**

Low educational level, lower sense of coherence, and mental distress (location of data collection in the psychosocial or general medical outpatient clinic) were associated with SUD. Those suffering from SUD seemed to be less aware of external stressors as SUD was also associated with low levels of reported post-migratory stress.

**Discussion:**

The association of SUD with psychological distress and lower education reaffirms the concept that some vulnerable groups are at a higher risk for substance-related difficulties. Strengthening the sense of coherence with targeted interventions might enable at-risk groups to cope better with forthcoming burdens and help with abstaining from current or future consumption.

## 1 Introduction

Displaced people are known to be particularly vulnerable to various kinds of health challenges (1, 2). Adverse and life-threatening events of all kinds can occur before, during, and after the displacement and pose a threat to the mental health of the fleeing individual (3, 4). Recent studies have reported prevalence rates of up to 39.8% for depressive symptoms, 40% for anxiety, and 37% for PTSD among refugees and asylum seekers in Germany (5, 6). Aside from adverse events, contextual factors, such as an excessive duration of the flight or an existing language barrier, can add onto the mental burden (7, 8). Whether it be an attempt to cope with the mental burden or due to a higher general vulnerability, displaced people certainly have an elevated risk of developing substance use disorders (SUD) (9).

Despite the high prevalence of mental health problems among populations of displaced people and a presumably high susceptibility for the use and abuse of psychoactive substances, the knowledge about SUDs within the population remains scarce (10). The available studies are limited and likely underestimate the problem, since the stigma surrounding SUDs can lead to underreporting and non-engagement among the participants (10, 11). We know from general population samples that young age (12), male sex (13), low educational levels (14), absence of religion (15), and not having children (16) can contribute to the risk of developing a SUD. In a flight- related context, young age (17) male sex (18), and low educational levels (19) have found to be risk factors for developing a SUD. The association between traumatic events and SUD is well documented (18, 20, 21) and fleeing individuals seem to be more susceptible to SUD when fleeing alone or when the escape itself lasts for a longer period of time (22). However, there is still a need to gain a better understanding of potential protective effects for SUD in populations of displaced people.

There are various factors that might contribute to the development of SUD or, alternatively, protect subjects from becoming addicted. With data from general population samples, we know that reducing the overall distress level (23) and healthy habits of emotion regulation - with cognitive reappraisal as the preferred mechanism of emotion regulation, rather than expressive suppression – are favorable (24). These positive effects have also been observed in individuals with a strong sense of coherence, a concept that attributes positive resiliency effects to the feeling of manageability, comprehensibility, and meaningfulness regarding personal situations and life activities (25). The same effects have been found within refugee populations for emotion regulation (26) and populations of forcibly displaced people for sense of coherence (8). Whether the protective influence that these factors seem to have on mental health extends to SUDs in populations of displaced people remains unclear, since, to our knowledge, no study has investigated these effects.

Therefore, this study was designed in an effort to gain a better understanding of risk factors and potentially protective factors regarding SUD within populations of displaced people in Germany. In order for displaced people to obtain any legal status in Germany, they have to settle a claim for asylum. While the term ‘displaced people’ is used for individuals that had to leave their home in general, the term ‘asylum seeker’ is used for those displaced individuals arriving in Germany and registering for the asylum process. This comprises all registered displaced individuals arriving in Germany, regardless of the potential legal status they may be granted later (e.g., refugee status). Considering (1) the high vulnerability for substance use disorders among displaced individuals suffering from (psychological) distress, (2) the variety of potential influence factors possibly playing a role in the development of substance use disorders, and (3) the varying concepts of mechanisms possibly underlying substance use in displaced persons, the following research questions were established: (1) Are levels of substance use disorders elevated among asylum seekers with high (psychological) distress? (2) Which risk/protective factors influencing prevalence of substance use disorder can be identified? (3) Which mechanisms may play a role in substance use patterns (self-medication vs. exacerbation of pre-existing use), when did current users start to consume?

## 2 Methods

### 2.1 Medical health care in state reception centers and study setting

Applying for asylum in Germany involves a number of different steps, including medical examinations, formal registration, and interviews by state officials (27). In an attempt to facilitate the application process and increase the speed of asylum applications to be handled, initial reception centers have been established all over Germany. These centers handle multiple administrative steps in a single location. After applying and getting an initial medical screening (with the aim of detecting especially infectious or potentially severe diseases), access to medical care is usually sought out. However, in these camp-like settings, this is usually limited due to Asylum Seekers Benefits Act (28). The state reception center “Patrick-Henry-Village” in Heidelberg, Kirchheim (PHV) poses an exception, as more extensive medical care in an outpatient clinic setting is offered here. This outpatient clinic consists (amongst other things) of a psychosocial and a general medical outpatient clinic (29). The team of the psychosocial outpatient clinic is part of Heidelberg University’s Center for Psychosocial Medicine and is comprised of 3 psychologists, a specialist in psychosomatic medicine, and a psychiatrist. Patients can be admitted by the staff of the general medical outpatient clinic, be referred by social workers or state officials involved in the registration process or present themselves independently. In addition to clinical diagnostics, the services offered consist of the documentation of diagnoses and corresponding treatment recommendations as well as the implementation of brief interventions, stabilization exercises, and the prescription of acute medication (30). While staying in the camp and waiting for the asylum application to be processed, the applicants are hosted in shared flats within former military barracks (29).

### 2.2 Participants and eligibility

The data analyzed in this study was collected between January 2021 and May 2021 at the initial reception center for asylum seekers, ‘Patrick-Henry-Village’ (PHV), in Heidelberg, Baden-Württemberg. We invited registered asylum seekers in three different settings within the PHV to participate in the study: (1) individuals who consulted the psychosocial outpatient clinic (2) individuals who consulted the general medical outpatient clinic (3) individuals who had already registered for asylum and are living in accommodations in the PHV but have not consulted a medical practitioner within the PHV so far. The inclusion criteria consisted of language fluency in either Arabic, English, Farsi, French, German, Serbian or Turkish; an age of 18 years or older and the ability to consent. Exclusion criteria consisted of illiteracy, an age below 18 years, and inability to provide consent.

### 2.3 Study design

We used a cross-sectional study design with three different subgroups - invitation to participate in the study in (1) The PHV psychosocial outpatient clinic, (2) the PHV’s general medicine outpatient clinic, (3) accommodations within the PHV to compare rates of substance use disorder and, in a next step, assessed the connection between potential risk/protective factors and substance use outcome among those asylum seekers who are (1) suffering from a mental health impairment, (2) suffering from a general health impairment or (3) not suffering from a health-impairment currently.

### 2.4 Ethical approval

All participating asylum seekers have given their written informed consent according to the declaration of Helsinki. The ethics committee of the University of Heidelberg approved the study conduction (S-684/2017).

### 2.5 Recruitment

Potential participants were informed about the study and were invited to participate prior to consultation in the regarding outpatient clinic (subgroups 1 and 2) or in the shared accommodation rooms (subgroup 3). The asylum seekers were informed that neither their decision to (not) participate nor any part of the study itself would have an impact on their asylum process or their healthcare utilization process.

### 2.6 Data collection

The written information material, as well as the sociodemographic, psychometric, and substance use specific questionnaires, were provided in seven different languages (Arabic, English, Farsi, French, German, Serbian, Turkish). Surveys without previous translations were translated by professional translators into the respective language. After having filled out the consent form, participating asylum seekers answered a set of questionnaires on a tablet PC running EFS survey ® software. After answering sociodemographic, flight specific, and substance use related questions, participants were asked to give responses to several psychometric measures. The psychometric measures consisted of the Leipzig Short Scale of the Sense of Coherence Scale (SOC-9 L), the Symptom-Checklist-K-9 (SCL-K-9), and the Emotion Regulation Questionnaire (ERQ-10).

### 2.7 Measures

#### 2.7.1 Biographic variables

##### 2.7.1.1 Sociodemographic data

The first set of questions answered by the participants included sociodemographic information (gender, age, religion, number of children, education).

##### 2.7.1.2 Flight related data

In order to assess cultural background and flight related information, we asked participants about language proficiency in English or German, flight companionship, and the experience of potentially traumatizing events (PTEs). Specifically, we asked participants: ‘What where your reasons to flee?’, ‘What kind of stress were you exposed to during flight?’ and ‘What challenges are you exposed to in your accommodation?’. The variation of challenges throughout the process of fleeing was hereby accounted for by structuring the assessed PTEs in *pre-migratory* - asking for potential experience of: domestic abuse, witness of homicide, loss of family, threat to family, displacement, abuse/rape, war, lack of medical care, lack of economic prospects, lack of social prospects, discrimination, political persecution, torture, and other; *peri-migratory* - asking for potential experience of: hunger, duration of flight, danger to life, death of a relative, death of someone else, torture, abuse/rape, illegality, separation from family, imprisonment, and other; and *post-migratory* – asking for potential experience of: rejection of asylum application, noise/restlessness, hygiene, lack of privacy, discrimination, fear of violence, physical assaults, and absence of people one can trust. For all three categories, an adversity ratio, which divided the number of selected items through the total number of selectable items, was created for statistical analysis.

#### 2.7.2 Psychometric variables

##### 2.7.2.1 SOC-9 L (Sense of Coherence Scale, Leipzig Short Scale)

The SOC-9 L was used for assessment of participants’ sense of coherence in accordance to the concept proposed by Antonovsky (25). Antonovsky’s original research tool SOC-29 consisted of 29 items. Schumacher et al. (31) had later proposed the SOC-9 L, which is a shortened version with 9 items. The reduced number of items improved feasibility of application while showing good internal consistency (Cronbach’s alpha = 0.87) and high correlation with the original 29-item questionnaire. Individual items are rated on a 7-point Likert-type scale, the scale description depends on the item. For example, item 1 consists of the question: ‘Do you have the feeling that you are in an unfamiliar situation and do not know what to do?’ which can be answered on a range from 1 – titled ‘very seldom or never’ – to 7 – titled ‘very often’. For comparison, item 2 consists of the following: ‘When you think about your life, you very often…’, which again can be answered on a range from 1 –titled ‘feel how good it is to be alive’ to 7 –titled ‘ask yourself why you exist at all’.

##### 2.7.2.2 ERQ-10 (Emotion Regulation Questionnaire)

The ERQ-10 was used to assess strategies for emotion regulation, that is, to which extent a participant used strategies of reappraisal or strategies of suppression to regulate their emotions (32). The questionnaire includes 10 different items in the form of statements that must be evaluated. For an example of a statement that assesses for reappraisal strategies, see item 1: ‘When I want to feel more positive emotion (such as joy or amusement), I change what I’m thinking about’. An example for a statement that assesses suppression strategies can be found in item 2: ‘I keep my emotions to myself’. The evaluation of each item is done on a 7-point Likert-type scale, which ranges from 1 “strongly disagree” to 7 “strongly agree.” The questionnaire had previously shown good to acceptable internal consistency for reappraisal (Cronbach’s alpha = 0.82) and suppression (Cronbach’s alpha = 0.76) (33).

##### 2.7.2.3 SCL-K-9 (Symptom Checklist-90-Revised, short version)

The Symptom Checklist-90-Revised has proven to be a reliable tool for assessing overall levels of distress and has been broadly used in various settings, despite its considerable length (34). By selecting the 9 items that showed the highest correlation with overall distress level (derived from the original questionnaire as Global severity index, GSI-90), a more handy version was created for clinical use, the SCL-K-9 (35). This short version is correlates highly with the original version (*r* = 0.93) and shows good internal consistency (Cronbach’s alpha = 0.87) (35). In order to assess for a variety of symptoms, the participant is asked: ‘How much were you bothered or distressed over the past 7 days by…?’, followed by the symptom descriptions associated with each item. Examples of these descriptions are ‘…uncontrollable emotional outbursts’ (Item 1) or ‘…finding it difficult to start something’ (Item 2). The respondent answers on a 5-point Likert-type scale, which ranges from 0 (“not at all”), to 4 (“extremely”).

#### 2.7.3 Location of data collection.

As described above, data was collected in three different settings within the PHV: (1) The PHV’s psychosocial outpatient clinic, (2) the PHV’s general medicine outpatient clinic, (3) accommodations within the PHV. In order to compare the different subgroups, we introduced the variable location of data collection, which was included in the statistical analysis.

#### 2.7.4 Substance use related information

**Figure 1:**
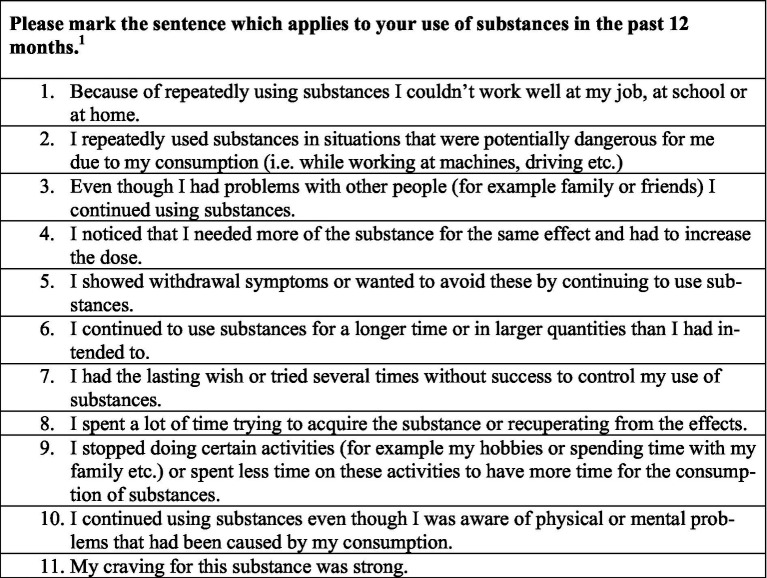
Assessment for substance use disorder according to DSM-5 (SUD-Screen).^1^ If a participant marked two or more items as applicable, this was considered a positive SUD-Screen.

Main outcome: Positive screening for substance use disorder (SUD-Screen). Participants who had reported consumption at some point were screened for SUD according to the definition by the *Diagnostic and Statistical Manual of Mental Disorders (DSM-5)* (36), which offers 11 different statements regarding substance use covering craving, tolerance, loss of control, risky behavior, and social impairment with potential scores ranging from 0 to 11 For the full set of items used, please see Figure 1. The presence of 2 or more symptoms during the last 12 months is defined as a substance use disorder and regarded as a positive SUD-Screen in the context of our study Details and secondary outcomes: all participating asylum seekers were asked for current or former use of substances; selectable categories were 1. Anxiolytic substances, tranquillizers, sleeping pills (Benzodiazepines) 2. Pain killers 3. Alcohol 4. Cannabis (Marihuana, Hashish, THC) 5. Stimulants (Amphetamines: Speed, Ritalin, Ice/Cristal Meth; Cocaine: Freebase, Crack, Speedball; Khat) 6. Opiates (Heroin, Morphine, Opium, Methadone, Codeine, Percodan, Demerol or others) 7. Hallucinogens (LSD, Mescaline, Psilocybin, PCP, Angel Dust, Ecstasy) 8. Others (i.e., steroids, solvents, and inhalants). In order for participants to specify which substance class they had used and during which time period, we structured the questions into four sections (before the flight, during the flight, after the flight, and never). This allowed us to determine the timeframe of consumption initiation among current users. If a participant reported using a certain type of substance, we followed up on this initial screening question with more detailed questions on the consumption patterns. In particular, we asked about period of consumption in years, means of acquisition, monthly days of consumption, and consumption motivation. We offered a variety of possible consumption motivation explanations to choose from, that can thematically be split into positive (4) – that is, the primary idea of consumption lies in pursuing a positive outcome, e.g., “to have fun” – and negative (9) – that is, the primary idea of consumption lies in avoiding a negative outcome, e.g., “to numb pain.” A ratio was formed by dividing the number of selected items through the number of available items in a category, resulting in a variable for each positive and negative motivation ranging from 0 (no item of this category describes the participants’ motivation for substance use) to 1 (all items in this category describe the participants’ motivations for substance use).

### 2.8 Statistical analysis

For statistical analysis, participants’ results were exported from EFS survey software ® as labeled Dataset and imported into IBM SPSS Statistics (Version 27). The dataset was then prepared for the subsequent analyses. This included a check on data quality, wherein we focused on straightlining. If a respondent continues to select the exact same response throughout a whole questionnaire, this might be an indication for low data quality (37). With the aim of identifying response-sets of low quality, we generated three auxiliary variables from the different psychometric questionnaires (SOC-9 L, ERQ, SCL-9). These variables reflect the individual’s response fluctuation throughout the questionnaires. If more than one of the three auxiliary variables showed a value of 0 (that is: the exact same answer throughout more than one set of questions), we excluded the participants data from further analysis. In order to find a balance between not including people who did not really respond to the questions thoughtfully, and also not excluding any valid responses (that by chance might have been very ‘symmetrical’ throughout a whole questionnaire, ‘straightlining’ a whole set of questions), we decided to set this cutoff. We then analyzed the data set for potential speeding, which could also be an indicator for low data quality (38). No indication for low data quality was found here, since none of the remaining participants’ respondence time was out of the two standard deviations-range on the low end. For an overview of sociodemographic, psychometric, flight-related and the main SUD-variable see Table 1. For the study variables included in our main statistical analyses, bivariate correlations were calculated using Pearson (rp), Spearman- (rs), and Phi- (rφ) coefficients (see Table 2), before introducing the variables into a hierarchic logistic regression analysis model separated into blocks (see Table 3) (For all additional descriptive statistics see Tables 4, 5). The blocks in the logistic regression analysis consisted of (1) sociodemographic variables (Gender, Age, Number of Children, Religion, Education), (2) flight-related variables (Flight companionship, Language proficiency, peri-migratory stress, pos-migratory stress), (3) psychometric variables (SOC-9 L, ERQ-10, SCL-K-9, 4) location of data collection as an indicator of (mental) distress. Pre-migratory stress correlated strong with during-flight stress as well as with post-migratory stress, causing a suppression effect in the regression analysis. Therefore, we excluded the variable pre-migratory stress from the regression equation. The hierarchical logistic regression allows us to isolate the specific impact of every variable on our main outcome variable (SUD-Screen) by simultaneously accounting for the effects of all other variables. Hierarchical logistic Regression Analysis was conducted by the glm-function of the statistic program R (39). No evidence of violation of multicollinearity were found (VIF < 1.92).

**Table 1:** Sample demographics, flight-related data, psychometric measures and SUD-Screen.

*N* = 238		*n* (%)	*M (SD)*	Range	Cronbach‘s alpha
Gender	Male	185 (77,7)			
	Female	48 (20,2)			
	Diverse	5 (2,1)			
Age (years)			29,06 (8,16)	18–68	
No. of children			0,93 (1,57)	0–8	
Religion	Islam	184 (77,3)			
	Christianity	25 (10,5)			
	Other	29 (12,2)			
Education	Not attended school	14 (5,9)			
	Attended school	106 (44,50)			
	Finished school	71 (29,8)			
	Finished university	47 (16,7)			
Language proficiency	No english/german	164 (68,90)			
	Speaking english/german	74 (31.10)			
Flight companionship	Traveling alone	161 (67.60)			
	Traveling in company	77 (32,40)			
Pre-migratory stress^1^			0,19 (0,14)		
Peri-migratory stress^1^			0,22 (0,16)		
Post-migratory stress^1^			0,23 (0,21)		
SOC-9 L-Score			39,96 (14,09)		0.81
ERQ-10 Cognitive reappraisal			3,36 (1,72)		0.86
ERQ-10 Expressive suppression			3,32 (1,65)		0.69
SCL-K-9-Score			15,95 (8,98)		0.85
Location	Psych. outpatient clinic	97 (40,8)			
	Gen. med. outpatient clinic	88 (37)			
	Residence in camp	53 (22,3)			
	
SUD-Screen positive^2^		43 (18.1)			

^1^Adversity ratios (calculated from the number of experienced potentially traumatizing events) are displayed as a quantification of pre-, peri- and post-migratory stress.

^2^Number of participants who met criteria for SUD (substance use disorder), according to DSM-5.

**Table 2:** Bivariate correlations.

Variable	1	2	3	4	5	6	7	8	9	10	11	12	13	14	15	16	17	18	19	20	21	22
SUD-Screen	–																					
Gender male	0.02	–																				
Gender female	−0.05	−0.94**	–																			
Gender diverse	0.08	−0.27**	−0.07	–																		
Age	−0.12	0.01	0.04	−0.13	–																	
Number of children	−0.13*	−0.17**	0.20**	−0.07	0.61**	–																
Religion Islam	−0.01	0.07	−0.05	−0.06	−0.05	0.06	–															
Religion Christianity	−0.02	−0.01	0.03	−0.05	0.01	−0.02	−0.63**	–														
Religion Other	0.01	−0.09	0.05	0.13*	0.04	−0.07	−0.68**	−0.12	–													
Education	−0.21**	0.16*	−0.14	−0.07	0.2**	−0.07	0.07	0.01	−0.08	–												
Flight companionship	−0.12	−0.17**	0.21**	−0.1	0.21**	0.41**	−0.01	0.03	−0.02	−0.11	–											
Language proficiency	−0.08	−0.01	0.02	−0.04	0.06	−0.05	−0.2**	0.13	0.11	0.25**	−0.06	–										
Pre-migratory stress	−0.03	0.16	−0.18**	0.05	−0.02	−0.03	0.11	−0.08	−0.05	−0.00	−0.04	0.02	–									
Peri-migratory stress	0.01	0.08	−0.13*	0.13*	−0.02	−0.03	−0.07	0.06	0.03	−0.03	−0.06	0.04	0.48**	–								
Post-migratory stress	−0.05	0.08	−0.07	−0.04	0.02	−0.04	0.06	−0.15*	0.07	0.01	−0.04	0.18**	0.34**	0.31**	–							
SOC-9 L-Score	−0.24**	0.14*	−0.12	−0.06	0.06	0.14*	0.22**	−0.04	−0.21**	0.06	0.06	−0.01	0.1	−0.05	−0.15*	–						
ERQ-10 Cognitive Reappraisal	0.01	−0.04	0.03	0.02	−0.11	−0.16*	−0.18**	0.16*	0.08	−0.02	−0.09	0.06	−0.12	0.01	−0.03	−0.07	–					
ERQ-10 Expressive Suppression	0.05	−0.11	0.08	0.11	−0.16*	−0.11	−0.13*	0.08	0.09	−0.07	0.05	0.1	−0.11	0.11	0.03	−0.17**	0.60**	–				
SCL-K-9-Score	0.25**	−0.09	0.06	0.09	−0.09	−0.16*	−0.06	−0.09	0.15*	−0.03	−0.12	−0.1	0.08	0.2**	0.21**	−0.57**	0.05	0.02	–			
Psych. Outpatient Clinic	0.21**	−0.13*	0.07	0.18**	−0.05	−0.11	−0.04	−0.09	0.11	−0.02	−0.21**	−0.04	−0.03	0.03	0.10	−0.28**	0.06	0.09	0.31**	–		
Gen. Med. Outpatient Clinic	−0.02	0.18**	−0.15*	−0.11	−0.02	−0.15*	0.02	0.08	−0.08	0.15*	−0.01	0.05	0.02	−0.02	−0.08	0.12	−0.12	−0.08	−0.17**	−0.64**	–	
Residence in camp	0.23**	−0.05	0.08	−0.08	0.07	0.29**	0.03	0.01	−0.03	−0.15	0.26**	−0.01	0.01	−0.02	−0.03	0.2**	0.06	−0.00	−0.17*	−0.44**	−0.41**	–

Pearson-, Spearman- and Phi- Coefficients with significant correlations at ^*^p < 0.05, ^**^p < 0.01.

**Table 3:** Logistic regression analysis – independent variables’ variance explanation of main outcome (positive SUD-Screen according to DSM-5 criteria).

	Step 1			Step 2			Step 3			Step 4			
	AOR	(95% CI)	*p*(Wald’s test)	AOR	(95% CI)	*p*(Wald’s test)	AOR	(95% CI)	*p*(Wald’s test)	AOR	(95% CI)	*p*(Wald’s test)	Power (1–β err prob)
Gender	1.01	(0.49,2.07)	0.989	0.97	(0.48,1.99)	0.941	0.73	(0.34,1.57)	0.426	0.68	(0.3,1.5)	0.338	0.264
Age	1.0	(0.94,1.06)	0.94	1.00	(0.94,1.06)	0.99	1.00	(0.94,1.07)	0.921	0.99	(0.93,1.06)	0.843	0.086
No. of children	0.72	(0.5,1.03)	0.074	0.78	(0.54,1.12)	0.18	0.83	(0.56,1.22)	0.345	0.91	(0.6,1.38)	0.661	0.173
Religion Islam	1.23	(0.38,3.96)	0.727	1.44	(0.4,5.14)	0.572	1.6	(0.4,6.31)	0.503	1.55	(0.38,6.37)	0.544	0.445
Religion other	1.13	(0.27,4.79)	0.87	1.37	(0.3,6.35)	0.688	0.83	(0.16,4.3)	0.824	0.62	(0.11,3.59)	0.597	0.498
Education	0.51	(0.33,0.8)	0.003	0.52	(0.32,0.83)	0.006	0.48	(0.29,0.78)	0.003	0.41	(0.25,0.69)	< 0.001	0.889
Flight companionship				0.58	(0.24,1.37)	0.212	0.57	(0.22,1.44)	0.233	0.78	(0.29,2.12)	0.63	0.216
Language proficiency				0.83	(0.35,1.96)	0.679	0.92	(0.36,2.34)	0.863	1.11	(0.42,2.9)	0.835	0.010
Peri-migratory stress				5.41	(0.61,48.01)	0.13	4.27	(0.39,46.84)	0.235	4.76	(0.38,59.88)	0.227	0.434
Post-migratory stress				0.3	(0.05,1.98)	0.211	0.1	(0.01,0.88)	0.038	0.1	(0.01,0.93)	0.043	0.580
SOC-9 L-Score							0.96	(0.93,0.99)	0.021	0.96	(0.93,1)	0.048	0.905
ERQ-10 Cognitive reappraisal							0.83	(0.61,1.13)	0.237	0.86	(0.62,1.18)	0.344	0.523
ERQ-10 Expressive suppression							1.16	(0.86,1.57)	0.327	1.14	(0.83,1.56)	0.414	0.420
SCL-K-9-Score							1.05	(0.99,1.11)	0.084	1.04	(0.99,1.1)	0.134	0.739
Gen. med. outpatient clinic										0.75	(0.31,1.81)	0.525	0.264
Residence in camp										0.06	(0.01,0.49)	0.009	1.000
Hosmer and Lemeshow	7.36			8.18			2.90			8.63			
Goodness of fit test, Chi^2^, (*p*), df = 8	(0.498)			(0.041)			(0.940)			(0.374)			
Likelihood ratio test for MLE method, Chi^2^, *p*	15.51			10.58			17.60			12.38			
	0.017			0.010			0.001			0.002			
McFadden R^2^	0.07			0.12			0.19			0.21			
Δ R^2^	0.07			0.05			0.07			0.03			
Log-likelihood	−104.68			−102.12			−92.09			−85.74			
AIC value	223.36			226.23			214.17			205.47			

**Table 4:** Number of people reporting substance use, sorted by time period and substance class.

*N* = 238	Never used	Before flight	During flight	Currently using
	*n* (%)	*n* (%)	*n* (%)	*n* (%)
Painkillers	157 (66.9)	35 (14.7)	33 (13.9)	46 (19.3)
Anxiolytics	173 (72.7)	30 (12.6)	30 (12.6)	31 (13.0)
Alcohol	192 (80.7)	27 (11.3)	22 (9.2)	22 (9.2)
Cannabis	207 (87.0)	23 (9.7)	17 (7.1)	8 (3.4)
Stimulants	226 (95.0)	5 (2.1)	7 (2.9)	2 (0.8)
Opiates	233 (97.9)	1 (0.4)	4 (1.7)	1 (0.4)
Hallucinogens	225 (94.5)	9 (3.8)	4 (1.7)	5 (2.1)
Others	236 (99.2)	1 (0.4)	0 (0)	1 (0.4)

**Table 5:** Timeframe of consumption onset among current users.

*N* = 238	*n* (%)		*n*	(%)
Painkillers	46 (19,3)	start before setting out	14	(30,40)
		start during the flight	4	(8,70)
		start after the flight	28	(60,90)
Anxiolytics	31 (13,0)	start before setting out	10	(32,30)
		start during the flight	2	(6,50)
		start after the flight	19	(61,30)
Alcohol	22 (9,2)	start before setting out	10	(45,50)
		start during the flight	4	(18,20)
		start after the flight	8	(36,40)
Cannabis	8 (3,4)	start before setting out	6	(75,00)
		start during the flight	1	(12,50)
		start after the flight	1	(12,50)
Hallucinogens	5 (2,1)	start before setting out	2	(40,00)
		start during the flight	0	(0,00)
		start after the flight	3	(60,00)
Stimulants	2 (0,8)	start before setting out	0	(0,00)
		start during the flight	0	(0,00)
		start after the flight	2	(100,00)
Opiates	1 (0,4%)	start before setting out	1	(100,00)
		start during the flight	0	(0,00)
		start after the flight	0	(0,00)
Others	1 (0,4%)	start before setting out	0	(0,00)
		start during the flight	0	(0,00)
		start after the flight	1	(100,00)

## 3 Results

### 3.1 Attrition and sample composition

From January 2021 to May 2021, we invited a total number of 524 asylum seekers to participate in our study, of which 334 agreed to participate (Participation quota: 61.6%). Of these participants, 59 were not able to respond to all our questions (e.g., due to lack of time), which equals a drop-out of 17.7%. 275 participants completed the questionnaire (Termination quota: 82.3%). Screening the remaining participants’ data for low data-quality led to another 37 people to be excluded from the analysis. These 37 participants showed straightlining-patterns, responding with a single value to all questions within one set of items on more than one psychometric tool (out of SOC-9 L, ERQ and SCL-K-9). This left us with a remaining number of *N* = 238 individuals’ data available for analysis, out of which *n* = 97 had been recruited in the psychosocial outpatient clinic, *n* = 88 in the general medicine outpatient clinic, and *n* = 53 within the general residences in the PHV (see Figure 1).

### 3.2 Sociodemographic

The sample characteristics are depicted in Table 1: the population was rather young (M age = 29.1 years, SD = 8.16) and predominantly male (*n* = 185, 77.7%).Educational levels ranged from not having a degree, that is “not attended school” (*n* = 14, 5.9%), and “attended school” (*n* = 106, 44.5%) to having “finished school” (*n* = 71, 29.8%) and having “finished university” (*n* = 47, 19.7%).

With regard to religious/spiritual beliefs, a majority (*n* = 184, 77.3%) described themselves as being Muslim. The second largest group was formed by Christians (*n* = 25, 10.5%) and a variety of other religious/spiritual orientations (Atheism, Judaism, Hinduism, Buddhism, “others”) was quoted and sub summarized in the category “others” (*n* = 29, 12.2%) for statistical analysis. The number of children ranged from 0 to 8 children (*M* = 0.93, SD = 1.57).

For the analyses of missing data (only two data points were missing), we first used Little’s *χ*2 test, which provided evidence for the assumption of missing completely at random (MCAR). Missing values were therefore imputed by the “mice” package (40) of the statistic program R (41).

### 3.3 Flight related data

Table 1 summarizes results of other flight-related variables: language proficiency in either English or German was not available to the majority (*n* = 165, 68.9%), while a minority (*n* = 74, 31.1%) stated to be able to use at least one of the aforementioned languages. 161 individuals (67.6%) traveled alone, while 77 individuals (32.4%) stated to have traveled in company. An additional overview of the experienced PTEs, structured by pre-, peri- and post-migratory events, is depicted in Table 6. Adversity ratios for the pre-, peri- and post-migratory phase were calculated from the amount of experienced PTEs for the regarding participant, depicted as pre-, peri- and post-migratory stress in further analyses.

**Table 6:** Experience of potentially traumatizing events.

Pre-migratory stress	*n*	(%)	Peri-migratory stress	*n*	(%)	Post-migratory stress	*n*	(%)
War	102	(42,90)	Danger to life	154	(64,70)	Noise, restlessness	79	(33,20)
Political persecution	68	(28,60)	Hunger	90	(37,80)	Fear of violence	76	(31,90)
Torture	65	(27,30)	Duration of flight	51	(21,40)	Rejection of asylum application	61	(25,60)
Threat to family	57	(23,90)	Imprisonment	50	(21,00)	Hygiene	60	(25,20)
Discrimination	51	(21,40)	Separation from family	48	(20,20)	Absence of people you can trust	57	(23,90)
Lack of medical care	41	(17,20)	Torture	46	(19,30)	Lack of privacy	52	(21,80)
Loss of family	38	(16,00)	Death of a relative	31	(13,00)	Discrimination	29	(12,20)
Lack of economic prospects	35	(14,70	Abuse/Rape	29	(12,20)	Physical assaults	20	(8,40)
Displacement	32	(13,40)	Illegality	28	(11,80)			
Lack of social prospects	32	(13,40)	Death of someone else	17	(7,10)			
Abuse/Rape	27	(11,30)	other:	25	(10,50)			
Domestic abuse	25	(10,50)						
Witness of homicide	25	(10,50)						
other:	39	(16,40)						

### 3.4 Psychometric data

The participants’ scoring in psychometric measures of sense of coherence (SOC-L9: M = 39.96, SD = 14.09), of emotion regulation ERQ-9 (Cognitive Reappraisal: M = 3.36, SD = 1.72; Expressive Suppression: M = 3.32, SD = 1.65), and of general symptom load (SCL-K-9: 15.95, SD = 8.98) are depicted in Table 1. Since we used questionnaires in different languages, we assessed for internal consistency by calculating Cronbach’s alpha. The results were satisfactory: SOC-L9 with Cronbach’s alpha = 0.81 (reference publication (31): Cronbach’s alpha = 0.87), for ERQ-10 Cognitive Reappraisal with Cronbach’s alpha = 0.86 (reference publication (33): Cronbach’s alpha = 0.82), ERQ-10 Expressive Suppression with Cronbach’s alpha = 0.69 (reference publication (33): Cronbach’s alpha = 0.76) and SCL-K9 with Cronbach’s alpha = 0.85 (reference publication (35): Cronbach’s alpha = 0.87).

### 3.5 Substance use

#### 3.5.1 Hierarchical logistic regression analysis

The results of the bivariate correlation are shown in Table 2 and results of the hierarchical regression analysis in Table 3. In the logistic regression, results of Hosmer-Lemeshow-Test and Likelihood-Ratio-Test show good model fit. 24% of variance could be explained by the examined variables. Most variance could be attributed equally to the influence of block 1 and block 3, that is, sociodemographic and psychometric variables. In the final step, we identified lower education, lower post-migratory stress, lower Sense of Coherence scores, and the psychosocial or general medical outpatient clinic as the location of data acquisition as significant correlates of the PAS use disorder prevalence. These findings may help answer our first research question, as they indicate that participants who were seeking medical/psychological help were at higher risk of being affected by SUD. Additionally, the identification of level of education, of post-migratory stress, and Sense of coherence as relevant factors regarding the risk of being affected by SUD constitutes an answer to our second research question. Other variables’ directional associations were according to our expectations based on the available literature but remain non-significant.

#### 3.5.2 Secondary outcomes

Results of the additional variables on substance use, describing numbers of active consumers for the regarding period and the timeframe of consumption onset among current users, are depicted in Tables 4, 5. Currently most used substances were Painkillers (46 participants, 19.3% of all), Anxiolytics (31 participants, 13,0% of all), and Alcohol (22 participants, 9.2% of all). Of those who were currently using these substances, the majority had started consuming during or after displacement This could be observed with painkillers (32 out of 46 participants (69.6%) started during or after displacement), Anxiolytics (21 out of 31 participants (67.7%) started during or after displacement) and Alcohol (12 out of 22 participants (54,5%) started during or after displacement). These results form the basis of our answer to the third research question of this study and will be discussed under point 5.3 (Substance use dynamics in the course of fleeing).

## 4 Discussion

### 4.1 The (mental) burden of SUD

We attempted to assess the extent to which (mental) distress is associated with SUD among asylum seekers and which sociodemographic, flight-related or psychological factors might play role in this context. Apart from that, we hoped to contribute to understanding displacement-related substance use dynamics by investigating consumption activity throughout different phases of the fleeing process.

While SUD rates were very low among those who were approached in the residence setting, the amount of people suffering from SUD was far higher among those who were approached in the medical outpatient clinics, both in the psychosocial and the general medical outpatient clinic. In particular, the burden of SUD seems to affect those who are already struggling with other health problems. Considering the well-known association between psychological distress and SUD (18, 42, 43), and the high prevalence rates for PTSD among populations of asylum seekers and refugees in institution-based samples (levels of PTSD were found to be at least as high as 20% (44), here) it is not surprising to find elevated levels of SUD among those who seek help for their psychological needs. Although the research regarding SUD among asylum seekers in Germany is limited, our findings align with what is known from previous research. One study screened for alcohol use, drug use, and extensive use of medications in a clinical setting of mentally distressed people, with 7.5% of participants reporting alcohol use, 6.6% reporting drug use and 22.8% extensive use of medication (45). A second study focused on the experiences of medical professionals working at a psychosocial outpatient clinic and included an overview of the most common clinical diagnoses. A considerable amount (17.4%) of the patients there had a SUD diagnosis (30). Combining the information from these earlier studies with the theoretical background of trauma related SUD, it seems safe to say that our findings reflect the outlines of a real problem. There are various possible reasons as to why levels of SUD were also elevated among those reaching out to a general practitioner. The process of somatization, leading to physical symptoms in an individual suffering from mental distress, might initially be the most important reason why a mentally distressed individual might end up seeing a general practitioner (46). Headaches and other (unspecific) pain syndromes have, by far, been the most frequently used diagnoses in medical ambulances for asylum seekers in the past (47). Similarly, fear of stigmatization or differing concepts of mental health (46–48) might lead to an individual seeing a general practitioner rather than a psychologist. These considerations explain the elevated numbers of SUD among people suffering from general health problems as a reflection of general (mental) distress.

We do not know how many Asylum seekers with SUD receive sufficient treatment, but we know that many of the affected people present themselves to clinicians at some point, may it be a psychosocial or a general medical clinic. While contact may not be made under the agenda of a SUD treatment, the strongly contrasting findings from the general residential homes (almost no SUD) and the outpatient clinics suggest that almost all the affected individuals at least get in contact with a medical institution. Considering the high rates of SUD prevalence that our findings suggest in these clinical/institutional populations, it appears reasonable to consider the implementation of a low-threshold screening into clinical practice to help identify affected people and offer them treatment options. In light of the fact that many people do not mention existing substance use in medical consultations for fear of negative consequences with regard to the asylum procedure, there are some arguments in favor of introducing a short screening tool. A potential short screening tool could be similar in structure to those that are commonly used to assess for other mental disorders like the GAD-2 for anxiety (49), PHQ-2 for Depression (50) or PC-PTSD-5 for PTSD (51) and could even be used in combination with those. It could consist of a filter question regarding the experience of substance use and a subsequent question containing the items of the DSM-5 definition for SUD, similar to the method used in our study. A very recently published study suggests a comparable approach and reports on the development of a screening tool called RAS-MT screener (52). It was designed by selection of items that assess for the most common as well as the most severe mental health conditions reported in populations of displaced people. A great advantage of the developed tool is the variety of disorders that are assessed for and its good transcultural validity. This is particularly important given the continuous change in countries of origin of displaced individuals as conflicts develop in different regions. The results from the screening tool were compared to clinical diagnoses by trained physicians and showed a satisfactory sensitivity rate of 74%. Another approach would be to adapt substance use related questions from the structured clinical interview, SCID (45, 53).

### 4.2 Risk and protective factors

From the sociodemographic variables we analyzed, it was the educational level that showed a significant negative correlation with SUD, a finding that is in accordance with earlier research (14, 19, 54). Although the relationship between different sociodemographic risk factors seems to be complex (14), it might be helpful for the development of possible interventions to keep in mind the role education plays. It seems important to make potential interventions not only culturally sensitive, but also accessible to everyone, regardless of their education, especially since *understanding* SUD as a treatable condition seems to be perceived as one key factor for successful interventions (55, 56).

The observed results regarding flight specific influences were slightly more difficult to understand. We found a significant correlation between post-migratory stress and SUD, yet not in the expected way. Various authors consistently reported they had identified post-migratory stress as a harmful factor adding onto the mental health burden and deteriorating health outcomes (21, 57–61). However, our results showed that people who reported more post-migratory stress were less likely to be suffering from a SUD. While this finding is divergent to the result we expected from literature research, the key to understanding the depicted result might lie within the fact that the assessed levels of post-migratory stress do not necessarily represent the actual number of external stressors affecting the individual, but rather the *perceived* number of stressors. This is an important difference, considering that individuals who may use substances in an attempt to alleviate psychological distress are likely to use substances with a dampening effect (e.g., opiates, benzodiazepines) (62), an effect that might also be able to influence perception of external stressors. While the reduction of tension and distress might be a welcomed effect of the used substance and help the individual to “escape the past” (48), the dampening effects might also lead to an effect of *escaping the presence*, with a reduced awareness for potentially disturbing or challenging external factors. This finding might not be new, but it can be considered an important reminder that those who are suffering from a SUD might be in need of additional support, as affected individuals might partly be unaware of external stressors and potentially harmful conditions. Since we found a significant negative correlation between Sense of coherence and SUD, it makes sense to look at how it is possible to strengthen the individuals’ feeling of meaningfulness, comprehensibility, and manageability (31). Based on the salutogenic model of Antonosky (25), this could work through mobilization of personal resources and promotion of reflection within stressful situations (56). Comprehensibility, such as understanding the concept of mental health and SUD as a treatable disorder, and manageability, acknowledging SUD as a disorder that can be worked on with a psychologist, have also been identified in recent research as relevant factors for a culture sensitive treatment of SUD (55).

The treatment of SUD is always challenging and becomes even more difficult with the co-occurrence of other mental health problems like PTSD or depression (43, 63). From what we know through qualitative research on the experiences of medical personnel, handling SUD-patients is perceived as one of the most challenging tasks in working with displaced people (30). The risk/protective factors we identified indicate key factors to be considered for the conceptualization of interventions. Making sure that potential interventions are easy to access (no higher education should be required), educative (trying to acknowledge SUD as a treatable disorder), and make use of the Sense of coherence model (for example through focusing on self-efficacy experiences) might be the key to make this task somewhat more feasible.

### 4.3 Substance use dynamics in the course of fleeing

As the data on consumption activity during the different timeframes (pre-, peri- and post-migratory) was not included in the main statistical analysis, we are not able to make statements on any potential statistical significance of the observations that we depicted in Tables 4, 5. Nevertheless, we choose to include this descriptive presentation of observations with the hope to contribute to understanding substance use dynamics as well as the susceptibility and development of SUD in a flight related context. Traditionally, some authors have argued that post-flight SUD might have its roots in the flight-related exacerbation of pre-existing substance use (18), while others regarded substance use as an attempt to cope with traumatic events in the sense of self-medication (64–66). Newer models try to consider both of those aspects and additionally try to take into account the influence of other psychological conditions or legal and social circumstances (67). In an attempt to understand the development of SUD and establish targeted interventions, it is not only necessary to identify the factors that contribute to SUD susceptibility, but also to identify the vulnerable timeframe, in which substance intake starts. From the people that used Painkillers in our sample, 69.6% had started using those during or after the flight. From the people that used Anxiolytics, it was 67.8% that started after leaving their home. These results suggest that the flight itself represents significant psychological and physical demands that refugees are counteracting with drugs. Although those evaluations are not detailed enough to draw final conclusions, they indicate that a relevant number of people start using substances during or after flight. We sincerely hope that more research can be done to identify vulnerable phases in which substance use commonly starts so that preventive measures could be established in the most relevant settings.

### 4.4 Strengths and limitations

One of the most frequently named reasons not to participate in our study was the lack of reading and writing skills. While the data collection via tablet computers offered a cheap and feasible way of recruiting participants and gaining information about a rather large population sample, it may have contributed to selection bias by the exclusion of lower-educated individuals. The decision to obtain data through self-reporting questionnaires, as opposed to an interview with a qualified person, was a compromise between quality of data and feasibility that we considered reasonable. Yet, the results regarding the influence of education must be looked at and interpreted with these considerations in mind.

At various points during the interaction with (potential) participants, we explicitly stated that no response from our survey would have any impact on an ongoing asylum process. However, this was one of the most frequently expressed concerns our researchers heard, which suggests that this uncertainty might have had an influence on the reported results or the willingness to participate. Conversely, a considerable number of participants still reported substance use and screened positive for SUD, which suggests that the assessment was indeed effective, despite the difficult setting.

Looking at our sample, it is clear that participants were rather young (Mean age: 29.1 years) and predominantly male, with 77.7% of participants being men. While not being as pronounced, imbalance regarding the gender distribution can also be found in the overall population of asylum seekers in Germany in the year of concern [59.1% men in 2021, (68)]. This tendency is even stronger within the age-groups from 18 and 40, where up to 70% of asylum seekers were men. A similar phenomenon can be observed regarding the age structure. While data on the general population of asylum seekers in Germany does not allow the calculation of a mean age as publications are structured by age groups, the data reveals that 76.2% of all adult asylum seekers were between the age of 18 and 40 (68). Consequently, a sample that is rather young and predominantly male may be considered favorable, as there is a resemblance regarding the sociodemographic structure with the overall population of asylum seekers in Germany. However, findings might be different within populations of different age and gender distribution.

Furthermore, it must be mentioned that post-migratory stress was assessed for by the number of PTEs the individual was exposed to (see 3.7.1). While all of the listed PTEs are grave, there can of course be differences regarding the (perceived) intensity of different events. While a person might have only experienced one PTE, they may have perceived that as far more severe than somebody who was exposed to three PTEs of a lesser intensity. Adding onto the considerations regarding the interpretability of the influence of post-migratory PTEs is the result from the post-hoc power analysis we performed, as the calculated power was relatively low at 0.58.

Lastly, while hierarchical logistic regression is a valuable statistical tool for individually assessing the influence of selected variables, it does not inherently address the intrinsic limitations of cross-sectional studies that come through data collection at a single point in time. Due to the design of the study as a cross-sectional study, when analyzing the results, it must be noted that the identified relationships can be bidirectional, and causality may not be conclusive.

## 5 Conclusion

The considerable sample size of 238 included participants, the inclusion of participants with different educational levels, religious beliefs and biographic experiences, the use of hierarchic logistic regression for the statistical analysis, and the availability of three subgroups with a differing burden of (mental) distress form the strengths of the study. We feel that the inherent limitations of a cross-sectional study are within a good balance with the advantages of the hierarchic logistic regression analysis, which allowed us to selectively assess the influence of specific variables. While the results of self-assessed questionnaires on topics surrounded by stigma may in some cases suffer due to non-respondance or under-reporting, we were able to document associations of (mental) distress levels with the burden of substance use on a significant level and identify risk factors that can act as promising starting points for future interventions.

## Data availability statement

The raw data supporting the conclusions of this article will be made available by the authors, without undue reservation.

## Ethics statement

The studies involving humans were approved by Ethics Committee of the Medical Faculty Heidelberg. The studies were conducted in accordance with the local legislation and institutional requirements. The participants provided their written informed consent to participate in this study.

## Author contributions

MS: Conceptualization, Investigation, Writing – original draft, Writing – review & editing. CN: Conceptualization, Supervision, Writing – review & editing. CZ: Conceptualization, Writing – review & editing. H-CF: Writing – review & editing. EN: Writing – original draft, Writing – review & editing.
