# Correction: The burden of substance use and (mental) distress among asylum seekers: a cross sectional study

**DOI:** 10.3389/fpsyt.2025.1708731

**Published:** 2025-11-11

**Authors:** Maximilian Solfrank, Christoph Nikendei, Catharina Zehetmair, Hans-Christoph Friederich, Ede Nagy

**Affiliations:** Department of General Internal Medicine and Psychosomatics, University Hospital Heidelberg, Heidelberg, Germany

**Keywords:** asylum seekers, refugee, substance use disorders, mental health, risk and protective factors

There was a mistake in **Table 1** as published. There was a typing error with the counts for Religion. Islam was listed as 183 (correct: 184) and Christianity as 26 (correct: 25). The percentages for Islam and Christianity were off by 0.4 percentage points (76.9% → 77.3%; 10.9% → 10.5%).

**Table 1:** Sample demographics, flight-related data, psychometric measures and SUD-Screen.

*N* = 238		*n* (%)	M (SD)	Range	Cronbach’s alpha
Gender	Male	185 (77,7)			
	Female	48 (20,2)			
	Diverse	5 (2,1)			
Age (years)			29,06 (8,16)	18-68	
No. of children			0,93 (1,57)	0-8	
Religion	Islam	184 (77,3)			
	Christianity	25 (10,5)			
	Other	29 (12,2)			
Education	Not attended school	14 (5,9)			
	Attended school	106 (44,50)			
	Finished school	71 (29,8)			
	Finished university	47 (16,7)			
Language proficiency	No english/german	164 (68,90)			
	Speaking english/german	74 (31,10)			
Flight companionship	Traveling alone	161 (67,60)			
	Traveling in company	77 (32,40)			
Pre-migratory stress^1^			0,19 (0,14)		
Peri-migratory stress^1^			0,22 (0,16)		
Post-migratory stress^1^			0,23 (0,21)		
SOC-9L-Score			39,96 (14,09)		0.81
ERQ-10 Cognitive Reappraisal			3,36 (1,72)		0.86
ERQ-10 Expressive Suppression			3,32 (1,65)		0.69
SCL-K-9-Score			15,95 (8,98)		0.85
Location	Psych. outpatient Clinic	97 (40,8)			
	Gen. med. outpatient Clinic	88 (37)			
	Residence in camp	53 (22,3)			
SUD-Screen positive^2^		43 (18.1)			

^1^Adversity ratios (calculated from the number of experienced potentially traumatizing events) are displayed as a quantification of pre-, peri- and post-migratory stress.

^2^Number of participants who met criteria for SUD (substance use disorder), according to DSM-5.

The correct **Table 1** appears below.

A correction has been made to the section **3 Results**, *3.2 Sociodemographic*. In the text describing the counts of different religions among participants, values were reported partly incorrect as they were deriving from the typographical error in **Table 1**. The number of participants identifying as Muslim (Islam) was reported as 1 too low (183 instead of 184), the number of participants identifying as Christians was reported as 1 too high (26 instead of 25), and the percentage value of Christians accordingly was too high (10.9% instead of 10.5%). In the “Others” category, the percentage value was reported as 0.4 too low (11.8% instead of 12.2%). The corrected text reads:

“With regard to religious/spiritual beliefs, a majority (*n* = 184, 77.3%) described themselves as being Muslim. The second largest group was formed by Christians (*n* = 25, 10.5%) and a variety of other religious/spiritual orientations (Atheism, Judaism, Hinduism, Buddhism, “others”) was quoted and sub summarized in the category “others” (*n* = 29, 12.2%) for statistical analysis”.

The original version of this article has been updated.

